# An Unsuccessful Case of Cynanche Trachealis, in Which Tracheotomy Was Resorted to

**Published:** 1830

**Authors:** 


Art. II.—*An unsuccessful case of Cynanche Trachealis, in which tracheotomy was resorted to. An extract from a letter to* E. Atlee, M. D. from John L. Atlee, M. D. of Lancaster, Pa. Communicated by Dr. Atlee.

Dear Uncle,—

My son William, was taken, on the 9th of January, with the premonitary symptoms of fever, which by the next morning was fully developed, and accompanied by the usual symptoms of Cynanche Tonsillaris. He had also a hoarse cough, indicating some affection of the larynx, which we did not think of much consequence, as he had been frequently troubled with it before. The fever continued three days, when it yielded to purgatives and antimonials, so that on the 13th he was so far convalescent as to amuse himself during the day and evening in playing about the chamber. On that night however, his sleep was restless and disturbed, and he would frequently start up in bed in great alarm. About three o'clock, on the morning of the 14th, we were awaked by alarming symptoms of suffocation, which however, subsided so completely before I was sufficiently awake to observe them, that I again lay down in security. These paroxysms recurred occasionally until day break, when his mother, in great alarm awaked me, and told me that she thought William had the croup. Finding her apprehensions to be well grounded, the most active measures were adopted, and Dr. Hume and Muhlenberg, were called in to my assistance. Bleeding, emetics, calomel and blisters were freely resorted to—assisted by seneka and the hive syrup, but although we were able to procure temporary relief from the paroxysms, they would recur at intervals of six or eight hours. He remained in this situation through the 14th and 15th, and on the night of the 15th just after midnight, a violent paroxysm came on, and before I could adopt any decided measures for his relief, he was on the point of suffocation. I sent immediately for the physicians and ran down stairs for my instruments, determined, if other means should be unsuccessful, to open the trachea. On my return he was gasping for breath in his mothers arms, just on the point of dying from suffocation. Under these trying circumstances, I cut through the integuments and completed the opening into the trachea, just as the physicians entered the room.

For a minute he lay apparently lifeless, he then opened his eyes and looked around him, at the same time breathing so softly and sweetly that I could scarcely realize that life was not extinct.

In about five minutes he had so far recovered, as to appear, with the exception of the wound, entirely well. The wound was kept patulous until an instrument could be procured to keep it permanently distended. The mucus was expelled by coughing through the artificial opening, and removed as it accumulated—he breathed softly and sweetly—his pulse and skin were natural. The larynx however remained closed, and he breathed only through the artificial opening—he was unable to make any sound and could only indicate his wants by the motion of his lips.

His physicians, with whom Dr. Carpenter was now associated, thought with me that there was a fair prospect of recovery, but on the following evening his breathing became more frequent with other febrile symptoms, followed by a paroxysm similar to that which had preceded the operation. It appeared to me as if a spasm had seized the trachea below the opening, as the whole trachea was so contracted that I could scarcely introduce a probe. I succeeded however, in doing so, opened the trachea, and immediately he expelled a firm coagulum of lymph which had formed in the trachea and bronchiæ. Temporary relief followed, but in a few hours the paroxysms returned and continued to recur until they terminated his existence about 5 o'clock on the morning of the 17th.

So distressing were the spasms, that I almost regretted the performance of an operation which only prolonged the dear childs sufferings. But it was one of the means which had been successful in other hands, and had I omitted it I should forever have regretted it. Indeed I have always reflected upon myself for having once before neglected to operate under similar circumstances.

*Lancaster, April 11th*, 1830.

*Remarks by the Editor.*—Few persons, we think, who have witnessed the agonizing struggle for breath in the last stages of croup, will hesitate to applaud the decision and firmness of a parent under these desperate circumstances, in resorting to an operation, although with a reasonable conviction that it could afford but temporary relief.

Several cases of croup are related by Dr. Jackson, in a late number of the American Journal of the Medical & Physical Sciences, in one of which, resembling the present in many particulars, the operation was performed at his urgent request. The operation was performed by Dr. Barton, at 1 P. M. A membrane was removed by the forceps which, when unfolded, exhibited the form of the trachea, its bifurcation and several of the ramifications of the trachea. In a short time almost every symptom of the disease vanished, and the most favorable anticipations were indulged; but in the evening the febrile irritation returned; a mucopurulent discharge took place from the opening which became more viscid, and so abundant that it was difficult to keep the opening free; the breathing became more and more laborious; and the patient died at 5 A. M. on the next day.

We shall present to our readers the appearance exhibited in the post mortem examination of the trachea and larynx with the remarks of the writer.

"*Trachea and larynx.* The interior of the larynx was completely incrusted over, with membraniform exudation; it lined the internal face of the epiglottis; the external was free of it; the rima glottidis was nearly closed by it. The ventricles of Morgagni, the ligaments, &c. were no longer apparent from the thickness of the membranous layer, spread over the interior of the larynx. This layer adhered firmly to the mucous membrane, but its surface was irregular, appearing as though pieces had been detached.

"The membraniform exudation extended through the trachea, and penetrated the bronchia, below its ramification, but did not enter the smaller divisions. At the ramification, the calibre of the bronchial tube was so much diminished as merely to admit a small quill. In the trachea, the exudation was closely adherent to the mucous membrane; in the bronchia it was detached and tapered off to a point. It could be separated, in the thickest portions, into layers, appearing to have been formed by successive exudations."

"The lower portion of the trachea and bronchia contained a fluid of the consistency and possessing the colour of cream; the tonsils and the posterior fauces were covered with a copious, thick, viscid mucus, strongly resembling albumen. The fluid of the trachea, I suspect came from this source, and the alteration of its colour, proceeded from its mixture with the air. I observed before death, that every act of inspiration produced a singular rattling sound. The copious secretion from the fauces, could not be expectorated, in consequence of the opening into the trachea, which destroyed the phenomenon of coughing, and it was drawn into the trachea by the effort of inspiration. Hence the windpipe was filled with it, respiration embarrassed, and suffocation ensued from its accumulation in the trachea and bronchia, with that of the bronchial secretions, in the lungs, from the impossibility of expectorating them by the loss of the power of coughing."

These cases appear to our author to justify the following conclusions:—

"1st. That inflammation of the tonsils is a frequent precursor to croup in children; and that it ought consequently always, in them, to be attentively watched, and should be treated very actively by the means best adapted to reduce inflammation."

"2d. That the membranous exudation may commence in the lower part of the trachea and the bronchia, extending upwards, and does not invariably arise in the fauces or larynx, and proceed downwards."

"3d. That the most prompt and decisive remedy for sanguine inflammation—that is, sanguineous depletion—is the only certain remedy, and should be resorted to in the first periods of the disease, so as to precede the membranous exudation."

"4th. That the membranous exudation having been once produced to any extent, little expectation of a recovery is to be entertained. When this result does occur, it is to be regarded as fortuitous, depending on some extraordinary circumstance, and which cannot be calculated as a probable event."

"5th. That when the membranous exudation has been thrown out in the larynx, trachea, and bronchia, the application of muriatic acid to the fauces, so highly extolled by Bretonneau, and of nitrate of silver, recommended by Dr. Mackenzie, from the local and limited impression they must necessarily make, can promise no beneficial operation—their influence cannot be extended to the surfaces from which the exudation takes place. This practice can be of utility only in rare cases, where the difficulty arises from an obstruction limited to the glottis and fauces."

"6th. That laryngotomy, or bronchotomy, is a useless operation, when the membranous exudation has actually occurred, and extends, as it mostly does, to the trachea and bronchia; or, when the inflammatory irritation of the respiratory mucous membrane is not subdued, and a free secretion from it is produced. The impossibility of coughing after the operation, prevents expectoration, the fluids accumulate in the trachea bronchia, and bronchial ramifications in the lung, causing, finally, suffocation. The only case in which the operation promises success, is an obstruction confined entirely to the glottis, without disease prevailing to any extent in the respiratory mucous membrane."

